# A possible role of *Drosophila* CTCF in mitotic bookmarking and maintaining chromatin domains during the cell cycle

**DOI:** 10.1186/s40659-015-0019-6

**Published:** 2015-05-27

**Authors:** Wenlong Shen, Dong Wang, Bingyu Ye, Minglei Shi, Yan Zhang, Zhihu Zhao

**Affiliations:** Beijing Institute of Biotechnology, No. 20, Dongdajie Street, Beijing, Fengtai District 100071 China; College of Life Science, Capital Normal University, 105 Xisihuanbei Road, Beijing, Haidian District 100048 China

**Keywords:** dCTCF, Cell cycle, Chromatin domains, Mitotic bookmarking, Bioinformatics

## Abstract

**Background:**

The CCCTC-binding factor (CTCF) is a highly conserved insulator protein that plays various roles in many cellular processes. CTCF is one of the main architecture proteins in higher eukaryotes, and in combination with other architecture proteins and regulators, also shapes the three-dimensional organization of a genome. Experiments show CTCF partially remains associated with chromatin during mitosis. However, the role of CTCF in the maintenance and propagation of genome architectures throughout the cell cycle remains elusive.

**Results:**

We performed a comprehensive bioinformatics analysis on public datasets of *Drosophila* CTCF (dCTCF). We characterized dCTCF-binding sites according to their occupancy status during the cell cycle, and identified three classes: interphase-mitosis-common (IM), interphase-only (IO) and mitosis-only (MO) sites. Integrated function analysis showed dCTCF-binding sites of different classes might be involved in different biological processes, and IM sites were more conserved and more intensely bound. dCTCF-binding sites of the same class preferentially localized closer to each other, and were highly enriched at chromatin syntenic and topologically associating domains boundaries.

**Conclusions:**

Our results revealed different functions of dCTCF during the cell cycle and suggested that dCTCF might contribute to the establishment of the three-dimensional architecture of the *Drosophila* genome by maintaining local chromatin compartments throughout the whole cell cycle.

**Electronic supplementary material:**

The online version of this article (doi:10.1186/s40659-015-0019-6) contains supplementary material, which is available to authorized users.

## Background

Referred to CTCF, the CCCTC-binding factor is a zinc finger protein highly conserved from *Drosophila* to human, and is the only known insulator protein in vertebrates [1, 2]. Initially discovered as a repressor of the chicken c-myc gene [3], CTCF is reported to be involved in many cellular processes, including transcription activation and repression, chromosome insulation, X-chromosome inactivation, DNA replication, and nucleosome positioning [4–8].

The many functions of CTCF can now be viewed in the context of genome-wide analyses. Researchers have identified hundreds of thousands of CTCF-binding sites across the genomes in different tissues of different species [9–12]. The widespread distribution of CTCF can be attributed to the interactions between the zinc finger domains of this protein and specific DNA sequences; CTCF can bind divergent sequences by using different combinations of its 11 zinc fingers [13]. The canonical CTCF binding motif is 20 bp [9]; however, using new technique and large-scale data, researchers identified a 33/34-mer two-part CTCF motif in mammals [11, 14]. CTCF-binding sites with larger motifs usually show stronger ChIP signal enrichment and are more conserved [11, 13, 14].

With the development of in vivo imaging techniques and molecular methods based on proximity ligation (3C, 4C, Hi-C, etc.) [15–18], emerging evidence suggests that genomes are dynamically organized at multiple structural levels, and that the hierarchical three-dimensional structure of chromatin is remarkably important for cellular function [19]. It is possible that CTCF, through using different combinations of zinc fingers, interacting with different protein partners, and the last but not the least, employing various post-translational modifications, could mediate extensive intra- and inter-chromatin interactions [7, 8]. Furthermore, research strongly suggests that CTCF clusters with other architecture proteins, and that CTCF-binding sites are enriched at topologically associating domain boundaries in mammalian and *Drosophila* genomes [20–22]. Thus, it is likely that CTCF plays a conserved role in chromatin domain organization. In addition, CTCF may be the main component of the heritable epigenetic system, regulating the interplay between DNA methylation, nuclear architecture, and lineage-specific gene expression.

Recently, there has been a growing interest in how the transcription program is re-established during mitosis. Several transcription factors, including CTCF, have been documented to remain bound to mitotic chromatin [23–29], and CTCF is also reported to function during the entire cell cycle [30].

However, changes of CTCF-binding sites during the cell cycle, and functions of this protein at different cell cycle phases, remain largely uncharacterized. Could CTCF act as mitotic bookmarkers that help establish, maintain and propagate the genomic topological organization during the cell cycle is also unknown. In this report, we analysed dCTCF binding site in *Drosophila* genome using public available datasets, and identified sites that are bound in interphase and mitosis, only during mitosis and only during interphase. Further, we found differences in conservation, binding motives, and GO enrichments among these three classes of dCTCF-binding sites. In addition, we observed that dCTCF-binding sites of the same class preferentially localized closer to each other, and were highly enriched at chromatin syntenic and topologically associating domains boundaries. Thus, dCTCF might contribute to the three-dimensional architecture of the *Drosophila* genome by maintaining local chromatin compartments throughout the whole cell cycle.

## Results

### Cell cycle phase-specific dCTCF binding sites

**Fig. 1:**
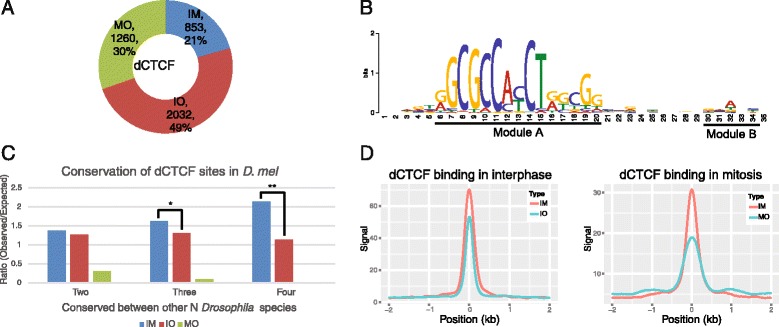
Characteristics of dCTCF-binding sites in interphase and mitosis. **a** Proportion of dCTCF-binding sites only in interphase (IO), mitosis (MO), or both (IM). **b** Analysis of motif enrichment of dCTCF-binding sites. **c** Obversed/Expected analysis of conserved dCTCF-binding sites. From left to right, bar plots show the ratio of the observed-to-expected number of conserved *D. melanogaster* specific binding events between at least N (2 ≤ N ≤ 4) *Drosophila* species (other species included *D. simulans*, *D. yakuba*, and *D. pseudoobscura*) (Fisher’s exact test, * *p* < 0.01, ** *p* < 0.0001). **d** Binding intensity of dCTCF-binding sites in interphase or mitosis. The x axes represent the distance from dCTCF-binding sites, and ‘0’ indicates the summit. Negative values indicate upstream and positive values indicate downstream of dCTCF-binding sites

We analysed *Drosophila melanogaster* ChIP data, and examined changes of dCTCF-binding sites during the cell cycle. Collectively, 4,145 dCTCF-binding sites were identified: 21 % of these sites were retained on chromatin during both interphase and mitosis, 49 % were present only during interphase, 30 % preferentially bound dCTCF only during mitosis, which were hereafter referred to as “interphase-mitosis-common” (IM) sites, “interphase-only” (IO) sites and “mitosis-only” (MO) sites, respectively (Fig. 1a, Additional file 1: Table S1).

Our de novo motif analyses revealed that dCTCF-binding sites were strongly enriched for the conventional dCTCF motif (Fig. 1b, module A) and also another slightly enriched motif (Fig. 1b, module B), which was previously reported in human and mouse [11]. We found this two-part motif was present in 60 % dCTCF IM sites and 51 % IO sites, but only 29 % MO sites. It was interesting that MO sites demonstrated low enrichment for this motif, but rather contained “GCW” repeats. By examining the GC content of all dCTCF-binding sites (Additional file 2: Figure S1B), we found dCTCF preferentially bound to GC-rich regions during mitosis. These data indicate dCTCF bound to DNA through different mechanism during interphase and mitosis phase. In addition, we identified a consensus Su(Hw) motif within CP190 IO sites, and a dCTCF motif within CP190 IM sites (Additional file 2: Figure S1C).

### dCTCF IM sites were tightly bound and highly conserved

During both interphase and mitosis, the average ChIP signal of dCTCF IM sites was significantly higher than that of IO or MO sites (Fig. 1d), which showed that IM sites were more tightly bound by dCTCF during the whole cell cycle. Meanwhile, motif analysis discovered that most dCTCF IM sites contained a two-module motif, indicating the relative high binding strength.

We performed the conservation analysis of dCTCF among different *Drosophila* species, and observed that dCTCF IM sites were significantly more conserved than IO sites (*p* < 0.0001, Fig. 1c). As no available data existed regarding MO sites in Drosophila species aside from D. melanogaster, it was hard to confirm whether MO sites were conserved between among different fly species.

### Different dCTCF-binding sites play different roles during the cell cycle

**Fig. 2:**
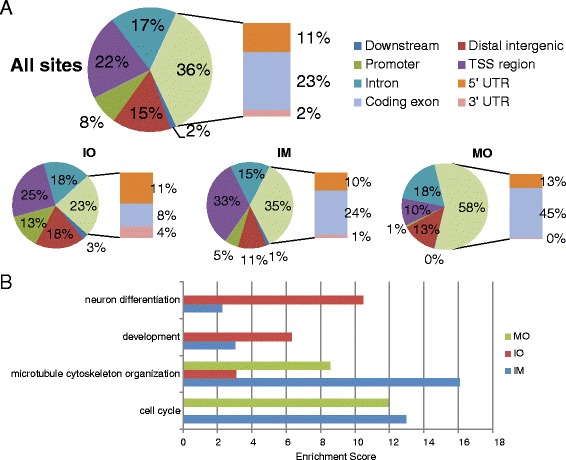
Functional annotation of dCTCF-associated genes. **a** Genome-wide distribution of dCTCF-binding sites relative to RefSeq genes. “TSS region” means a region within 200 bp of a transcription start site (TSS); “Promoter” means a region within 2 kb upstream of a TSS region; “Downstream” means a region within 2 kb downstream of a transcription termination site (TTS). **b** GO analysis of genes associated with cell cycle phase specific dCTCF-binding sites. Significantly enriched GO terms were manually grouped. The x axis shows enrichment scores calculated by DAVID

The high conservation of IM sites, paired with the high affinity of dCTCF for these sites, demonstrated the importance and indispensability of these dCTCF-binding regions during the cell cycle. To check if IM site confers particular function during the cell cycle, we first checked the genomic distribution of dCTCF-binding sites (Fig. 2a). While the proportions of IM and IO sites localized to the upstream (≤2 kb) of gene transcription start site (TSS) were almost the same, we determined that 33 % of IM sites localized to within 200 bp of TSS significantly higher than 25 % of IO sites (*p* < 0.01, Fisher’s exact test), suggesting that dCTCF bound to TSS regions more likely remained throughout the whole cell cycle. In contrast to IM and IO sites, MO sites were less enriched at TSS regions but more at exons (Fig. 2a). These are suggestive of that different class of dCTCF binding sites might exert different CTCF function in the cell cycle.

To gain insight into the functional differences of IM, IO, and MO sites, we performed GO analysis for genes associated with dCTCF-binding sites using DAVID [31] (Fig. 2b, Additional file 3: Table S2). Genes associated with IM sites showed a broad range of ontologies, indicating that IM sites were involved in many biological processes during the whole cell cycle. On the contrary, IO and MO showed distinct enriched ontologies. Particularly, IO sites were enriched at genes involved in neuron differentiation and development, which is possibly the result of the original ChIP data being collected from embryonic *D. melanogaster* Kc167 cells, a cell type in which dCTCF may be involved in the regulation of this major cellular process. MO sites were enriched at genes involved in microtubule cytoskeleton organization and cell cycle, indicating that these dCTCF-binding sites might play a key role in mitotic progression.

We subsequently evaluated co-occupancy status of dCTCF with other architecture proteins and epigenetic markers (Additional file 4: Figure S2). It was found dCTCF, BEAF-32, and CP190 co-localized mainly in gene TSS regions, and usually had high H3K4me3 and H3K27ac signals, which were also high around dCTCF MO sites distributed in coding exons during interphase. Although the intensity of these signals during mitosis was unknown, these results implied that dCTCF MO sites could be involved in specific regulatory functions during mitosis. Interestingly, BEAF-32 and CP190 were barely detectable near dCTCF MO sites. As dCTCF demonstrated more MO sites compared to BEAF-32 and CP190, it is likely that dCTCF functions differently from other insulators during mitosis.

### dCTCF-binding sites of the same class tend to maintain local compartments

**Fig. 3:**
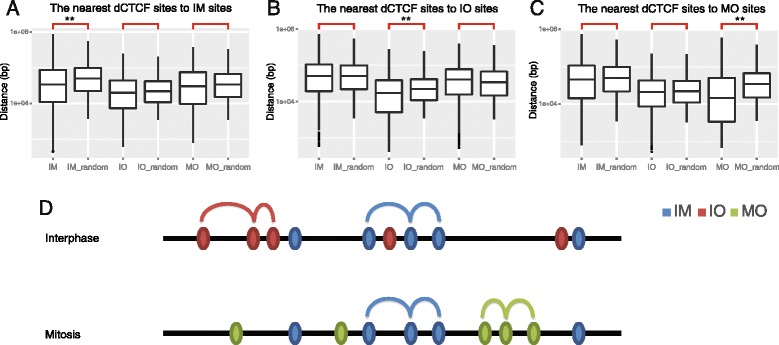
Non-random distributions of dCTCF-binding sites. **a**–**c** Shortest distances between specific dCTCF sites. Boxplots show the distribution of shortest distances between specific dCTCF sites; the suffix “_random” indicates control sets. Each panel (**a**, **b**, **c**) show the shortest distances of dCTCF sites to IM, IO, and MO sites, respectively. (Wilcoxon Test, ** *p* < 1e-8). **d** A proposed model of dCTCF loci in different phases of the cell cycle. Cell cycle phase specific dCTCF-binding sites of the same class were preferentially closer to one another, and appeared to establish cell cycle phase specific chromatin domains

Several researchers have related the diverse functions of CTCF to its ability to modify chromatin structures [7, 8]. We compared distances of nearest dCTCF binding sites to each dCTCF IM sites, and found distances between IM sites were significantly shorter than permutated chromosomes; while IO and MO sites, compared with permuted chromosomes respectively, showed no differences in distance to IM sites (Fig. 3a). This suggested that IM sites selectively clustered to IM site but not to IO and MO sites. Similar results were observed as for distances to IO and MO sites (Fig. 3b, c). These data indicated non-random distributions of dCTCF-binding sites, with the sites among the same category tend to cluster along chromosomes (Fig. 3d). Furthermore, this observation strongly excluded the possibility of binding leakage during mitosis, as dCTCF binds and leaves these sites non-randomly as some suggested for the other regulators such as FoxA1 [28].

**Fig. 4:**
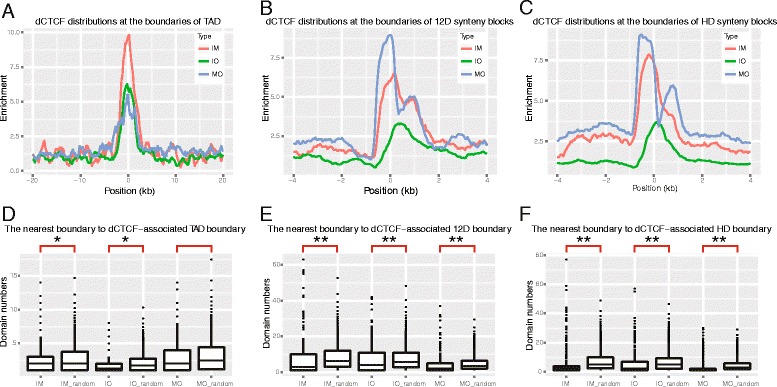
dCTCF-binding sites of the same class tend to maintain local domains. **a**–**c** The enrichment of dCTCF sites at domain boundaries. The abbreviation “12D” means regions of conserved synteny across 12 *Drosophila* species, and “HD” means syntenic blocks conserved between Human and *D. melanogaster*. The x axes represents the distance from specific dCTCF-associated boundaries. Negative and positive values indicate binding sites within and outside of domains, respectively. **d**–**f** Boxplots show the distribution of minimum domain numbers between specific dCTCF-associated boundaries. (Please notice that here we only plotted the IM/IM_random to IM boundaries, IO/IO_random to IO boundaries, and MO/MO_random to MO boundaries). The suffix “_random” indicates control sets. Boxplots are pairwise and marked by brackets. The distribution between normal and control sets are significantly different (Wilcoxon test, ** *p* < 1e-8, * *p* < 0.05)

To gain further insight into the differences between dCTCF sites, we examined whether certain dCTCF-binding sites were enriched at TAD boundaries. We confirmed enrichment of all class of dCTCF binding sites, especially IM ones (Fig. 4a). Then, we wondered if TAD boundaries containing specific dCTCF-binding sites also showed non-random distributions. We defined “IM boundaries” as domain boundaries containing dCTCF IM sites, with the same semantics for IO or MO boundaries, and determined distances between each specific domain boundaries by counting the minimum domain numbers. We found that IM boundaries tended to be closer to each other compared to the random sets (Fig. 4d), which also held true for IO sites, indicating that specific domains might be maintained by dCTCF-binding sites belonging to the same class. However, MO boundaries did not show the same phenomenon, possibly due to lack of TAD data pertaining to mitosis.

When we compared with syntenic blocks, we further found that dCTCF-binding sites were also in fact enriched at syntenic boundaries (Fig. 4b, c). These results showed that dCTCF-binding sites were enriched at not only higher-order spatial physical and functional domain boundaries, but also evolutionary conserved linear domain boundaries. With similar method to determine specific TAD boundaries afore mentioned, we finally checked distribution of IM/IO/MO syntenic block boundaries and random ones. Once again, we found that syntenic block boundaries belonging to the same group tend to be closer than random sets (Fig. 4e, f). With these findings, we hypothesized that dCTCF could contribute to the formation of boundaries at various chromatin domain levels, and that dCTCF-binding sites of the same class may maintain local chromatin compartments.

## Discussion

Here, we investigated the genome-wide distributions of three types of dCTCF-binding sites (i.e. IM, IO, and MO) in *D. melanogaster*. A large proportion of binding sites remained bound by dCTCF during mitosis. dCTCF IM sites usually contained two-module motifs, which was consistent with their stronger ChIP enrichment and higher conservation, implying that dCTCF IM sites might play important roles during the cell cycle and need to be retained during mitosis. Recent work in human cells revealed that, contrary to classical insulator function, most CTCF-binding sites mediate promoter-enhancer communication [32]. Coincidently, we found a larger proportion of IM than IO sites that located within TSS. It should be interesting to further examine whether TSS, and hence promoter-associated dCTCF IM sites actually promote rapid activation of gene expression during M/G1 transition.

The proportion dCTCF MO binding sites was much larger than that of recently reported mitotic bookmarking factors, for examples, MO sites for Myc, Polycomb Group (PcG) proteins, GATA1, FoxA1, and RBPJ accounted for only 0 % [25], 0 % [26], 9.9 % [27], 13.7 % [28], and 10.6 % [29], respectively; and this proportion is also higher than other architecture proteins in *D. melanogaster*, such as BEAF-32 and CP190, which were only 1 % and 4 %, respectively (Additional file 1: Table S1, Additional file 2: Figure S1A). Besides, these novel dCTCF MO sites demonstrated particular binding motives, agreeing with previous reports on GATA1 and FoxaA1, which also occupy mitotic-specific binding sites with different sequence features (compared to interphase binding sites) [27, 28]. As chromatin is condensed during mitosis, it is possible that the recognition pattern of certain transcription factors might change during this particular phase of the cell cycle. Such a substantial proportion of dCTCF MO sites and special binding motif promoted us to suspect particular roles these site might play in mitosis. Consequently, GO analysis implied that dCTCF MO sites were enriched at genes involved in microtubule cytoskeleton organization and cell cycle, indicating that these sites mediated dCTCF functions in mitotic progression, though the underlining molecular mechanism remains unclear. We observed that dCTCF preferentially bound to exon regions during mitosis, possibly due to reduction of open chromatin regions during mitosis. CTCF has been shown to bind to exons and plays a role in alternative splicing [33], whether dCTCF functions in a similar manner during mitosis in *Drosophila* remains to be an interesting open question.

Previous studies have suggested that dCTCF and Su(Hw) do not interact with one another directly, but rather co-localize with CP190 individually at distinct loci [34]. Our motif analysis identified a consensus Su(Hw) motif within CP190 IO sites, and a dCTCF motif within CP190 IM sites (Additional file 2: Figure S1C), as Su(Hw) was not typically present on chromosomes during mitosis [25], we postulated that CP190 recruited by Su(Hw) to chromosomes may be erased during mitosis while CP190 recruited by dCTCF may be maintained. Thus providing an extended view of these architecture proteins.

Several investigations have observed that a portion of the FoxA1, RBPJ, and H2A.Z binding peaks obtained by ChIP-seq appeared to shift in their genomic localization when mitotic and asynchronous interphase cells were compared. It was previously proposed that such peak shifting facilitated the rebinding of specific proteins to real target sites during the exit from M phase, which was accomplished by the partial maintenance or storage of mitotic bookmarking DNA binding proteins on mitotic chromatin. However, we did not observe a clear peak shifting phenomenon for dCTCF, which implied that the binding of dCTCF to MO sites might have functions other than the storage of this protein on mitotic chromatin. We suspected that dCTCF was maintained on mitotic chromatin by a different yet unknown mechanism. Further experiments, such as comparison of ChIP-seq results from different cell types of different cell cycle phases, will provide more clues about the binding mechanism and functions of CTCF during the cell cycle in different organisms.

Previous studies have demonstrated that partial dCTCF-binding sites serve as boundary elements. Our analysis confirmed this result, and found that dCTCF-binding sites were not only enriched at topological and functional domain boundaries, but also at evolutionarily conserved domain boundaries (i.e., syntenic blocks), which extended the definition of the “genomic domain”. We also found that cell cycle phase specific dCTCF-binding sites of the same class were preferentially bound closer to one another, and a similar result was observed for domain boundaries containing dCTCF-binding sites. Although MO boundaries of TAD did not show the same phenomenon, partially because of the lack of TAD data pertaining to mitosis, and recent Hi-C experiments suggested TADs were mostly lost during mitosis [35] in human cells. However, these results did not conclusively exclude the possibility of the existence of potential TAD boundaries. In vivo live imaging of potential domain boundaries during the cell cycle might help to rectify this issue. We proposed a model of dCTCF loci during different phases of the cell cycle (Fig. 3d), which indicated that cell cycle phase specific dCTCF-binding sites help to establish cell cycle phase specific chromatin domains. Further experiments such as cell cycle specific dCTCF knock down, or the degradation of this protein, might provide direct biochemical evidence for this model.

We proposed that dCTCF, by collaboration with other architecture proteins and sequence-specific transcription factors, may partially maintain local chromatin domain structures; such structures could be propagated through cell division, and facilitate the rapid re-establishment of chromatin structures and the re-activation of some genes during the M/G1 phase transition.

## Conclusions

We determine that dCTCF remains bound to chromatin throughout the cell cycle, and demonstrate several cell cycle phase specific sites with different sequence features. Each class of dCTCF-binding sites (i.e., IM, IO, and MO) plays different roles at different phases of the cell cycle. Specifically, IM sites appear to be involved in many biological processes during the entire cell cycle, whereas MO sites may play a key role only during mitosis. IM sites are also unique among dCTCF-binding sites, as they are highly conserved between *Drosophila* species, and are intensely bound by dCTCF. In addition to providing insight into the roles of dCTCF during the cell cycle, our results also indicate that dCTCF likely contributes to the formation of various chromatin domain boundaries, as cell cycle phase specific dCTCF-binding sites are closer to one another and appear to maintain cell cycle phase specific local compartments.

## Methods

### Datasets

The *Drosophila melanogaster* dCTCF, BEAF-32 and CP190 ChIP-seq data sets were downloaded from Yang et al. [25], (deposited in NCBI’s Gene Expression Omnibus (GEO) under accession numbers GSE32584). For cell synchronization, Yang et al. treated the cells with hydroxyurea and nocodazole. The enrichment of the mitotic and interphase cell populations showed 97–99 % purity. Histone modification data were from Kellner et al. [36], (GSE36374). dCTCF ChIP-seq data from other *Drosophila* species, used for the conservation analysis, were from Ni et al. [12], (GSE24449). Chromatin topologically associating domain boundaries were identified in Sexton et al. [21], and regions of conserved synteny across 12 *Drosophila* species were identified in Bhutkar et al. [37]. Syntenic blocks conserved between human and *D. melanogaster* were downloaded from Sinha et al. [38]. We only used genomic features data (e.g., genes and binding sites) available for chromosomes 2 L, 2R, 3 L, 3R, 4, and X.

### Motif analysis

For each insulator, we did de novo motif searching by MEME [39] using the top 1000 ChIP-seq-enriched binding site DNA sequences. And we also randomly chose 500 sequences from IM/IO/MO sites separately as candidate sets. To calculate the occurrence (%) of the two-part motif in each class of dCTCF-binding sites, we used FIMO tools from the MEME software suite. We downloaded motif matrix from JASPAR [40] for comparison.

### Conservation analysis

We mapped all the non-*D. melanogaster* species binding sites into the *D. melanogaster* genome with liftOver [41] using default parameters, except for a match of 0.5. The number of *D. melanogaster*-binding sites overlapping with each of the non-*D. melanogaster* liftOver binding sites was counted.

### Calculation of distances between specific dCTCF-binding sites or domain boundaries

We calculated the shortest distance between two specific sites for each dCTCF-binding sites, that is, we first chose one dCTCF-binding site as the bait, then calculated the distance between the bait and the nearest other site. We used the control sets by randomly shuffling the location of all sites along chromosomes. We termed any domain boundary containing dCTCF IM/IO/MO sites as “IM/IO/MO boundary”, and calculated the minimum domain numbers between specific boundaries. We permuted the dCTCF-binding sites at boundaries as control sets. The distribution of shortest distances, or minimum domain numbers of control sets, were computed 1,000 times followed by normalization.

## Additional files

Additional file 1: Table S1. Binding sites of dCTCF, BEAF-32, and CP190 for each class. Additional file 2: Figure S1. Characteristics of insulators binding events during interphase and mitosis. (A) Proportions of BEAF-32 and CP190 binding sites of each class. (B) GC content of dCTCF-binding sites DNA sequences. The x axis represents the distance from dCTCF-binding sites, and ‘0’ is the summit. Negative and positive values indicate upstream and downstream of dCTCF-binding sites, respectively. (C) Motif analysis of CP190-binding sites. The two upper motifs were de novo generated, and the two lower motifs were isolated from the JASPAR database. Additional file 3: Table S2. GO terms category enrichment identified by DAVID. Additional file 4: Figure S2. Occupancy of dCTCF cofactors at specific dCTCF-binding sites. Heatmaps showing signals of insulators and histone modifications at dCTCF-binding sites in interphase and mitosis. Each panel represents a 2 kb distance both upstream and downstream of the anchor dCTCF-binding sites. The suffixes “-int” and “-mit” mean ChIP signal in interphase and mitosis, respectively; histone modification data is unavailable for mitosis. The sites are ordered by different annotations.
