# Supplementary figures and images for: A possible role of Drosophila CTCF in mitotic bookmarking and maintaining chromatin domains during the cell cycle
Source: Biol Res. 2015 May 27;48(1):27. doi: 10.1186/s40659-015-0019-6 (PMC4485355; doi:10.1186/s40659-015-0019-6)

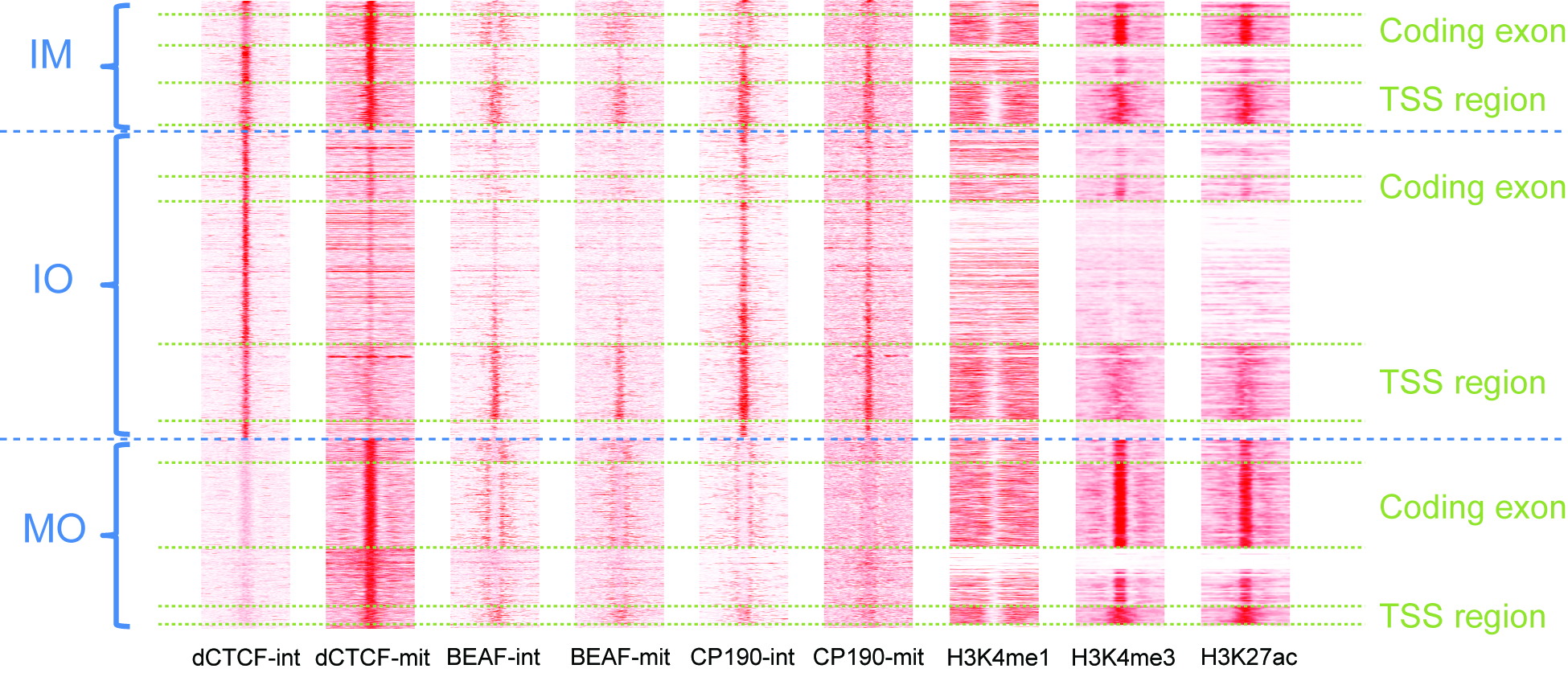

Supplement: Additional file 4: Figure S2. — Occupancy of dCTCF cofactors at specific dCTCF-binding sites. Heatmaps showing signals of insulators and histone modifications at dCTCF-binding sites in interphase and mitosis. Each panel represents a 2 kb distance both upstream and downstream of the anchor dCTCF-binding sites. The suffixes “-int” and “-mit” mean ChIP signal in interphase and mitosis, respectively; histone modification data is unavailable for mitosis. The sites are ordered by different annotations. [file 40659_2015_19_MOESM4_ESM.tiff]
